# Supplementary material for: Effects of Phenolic-Rich Pinus densiflora Extract on Learning, Memory, and Hippocampal Long-Term Potentiation in Scopolamine-Induced Amnesic Rats
Source: Antioxidants (Basel). 2022 Dec 19;11(12):2497. doi: 10.3390/antiox11122497 (PMC9774118; doi:10.3390/antiox11122497)
Supplement: Supplementary file 1 [file antioxidants-11-02497-s001.zip › antioxidants-2025323-supplementary.pdf]

antioxidants

Supplementary Files

Effects of Phenolic-rich *Pinus densiflora* Extract on Learning, Memory, and Hippocampal Long-term Potentiation in Scopolamine-induced Amnesic Rats

Kwan Joong Kim[1], Eun-Sang Hwang[2], Min-Jeong Kim[3], Chan-Su Rha[4], Myoung Chong Song[5], Sungho Maeng[2], Ji-Ho Park[2*] and Dae-Ok Kim[1,3*]

[1] Graduate School of Biotechnology, Kyung Hee University, Yongin 17104, Republic of Korea
[2] Graduate School of East-West Medical Science, Kyung Hee University, Yongin 17104, Republic of Korea
[3] Department of Food Science and Biotechnology, Kyung Hee University, Yongin 17104, Republic of Korea
[4] AMOREPACIFIC R&I Center, Yongin 17074, Republic of Korea
[5] Natural Products Research Institute, College of Pharmacy, Seoul National University, Seoul 08826, Republic of Korea
* Correspondence: jihopark@khu.ac.kr; Tel: +82-31-201-2916 (J.-H.P); DOKIM05@khu.ac.kr; Tel: +82-31-201-3796 (D.-O.K.)

Table of Contents

1. Materials and methods

1.1. Determination of total phenolic and flavonoid content

1.2. Determination of antioxidant capacity

2. Results and discussion

1.1. Total phenolic and flavonoid content

2.2. Antioxidant capacity

Table S1. Total phenolic and flavonoid content, and antioxidant capacity of *Pinus densiflora* Sieb. et Zucc. bark extract (PBE)

Figure S1. (A) Total ion chromatogram and (B) base peak chromatogram (filtered with MS^1) of *Pinus densiflora* Sieb. et Zucc. bark extract (PBE)

Figure S2. Typical fragmentation patterns for procyanidins (A) dimer and (B) trimer.

Figure S3. Typical fragmentation patterns for (A) 3-*p*-coumaroylquinic acid and (B) 5-*p*-coumaroylquinic acid.

Figure S4. Typical fragmentation patterns for (A) quercetin 3-*O*-rhamnoside and (B) quercetin-7-*O*-glucoside.

Figure S5. Typical fragmentation patterns for (A) procyanidin B type dimer and (B) procyanidin trimer via retro-Diels-Alder (RDA), heterocyclic ring fission (HRF), and quinone methide (QM) reactions.

Figure S6. MS^2 characteristics of *O*-glycosylated hydroxyflavonol.

Figure S7. Swimming time in each quadrant of trial acquisition in the Morris water maze test. The removed target platform (rTP) is located in target quadrant 3. Quadrants: Q1, quadrant 1; Q2, quadrant 2; Q3, quadrant; Q4 quadrant 4

1. Materials and methods

1.1. Determination of total phenolic and flavonoid content

The total phenolic content of the *Pinus densiflora* Sieb. et Zucc. bark extract (PBE) was measured using the colorimetric method with Folin-Ciocalteu's phenol reagent [1]. Two hundred microliters of PBE (100 mg/L) Two hundred microliters of PBE (100 mg/L) was mixed with 2.6 mL of deionized water. Then, 200 μL of Folin-Ciocalteu's phenol reagent was added to the mixture. At 6 min, 2 mL of 7% (*w*/*v*) Na_2CO_3 solution was added; at 90 min, the absorbance was measured at 750 nm using a spectrophotometer (SPECTRONIC 200; Thermo Fisher Scientific Inc., Waltham, MA, USA). The content of total phenolics was expressed as mg gallic acid equivalents (GAE)/g extract.

The total flavonoid content of the PBE was measured using the method of [2]. Five hundred microliters of PBE (100 mg/L) was mixed with 3.2 mL of deionized water. Then, 150 μL of 5% (*w*/*v*) $NaNO_2$ solution was added. After 5 min, 150 μL of 10% (*w*/*v*) $AlCl_3$ was added. At 6 min, 1 mL of 1 M NaOH was added, and absorbance was measured immediately using a spectrophotometer (SPECTRONIC 200; Thermo Fisher Scientific Inc.). The content of total flavonoids was expressed as mg catechin equivalents (CE)/g extract.

1.2. Determination of antioxidant capacity

The antioxidant capacity of PBE was determined using the ABTS and DPPH radicals [3] and expressed in mg vitamin C equivalents (VCE)/g extract. The ABTS radical solution was adjusted to an absorbance of 0.650 ± 0.020 at 734 nm using a spectrophotometer (SPECTRONIC 200; Thermo Fisher Scientific Inc.). The reaction between ABTS radicals and the PBE (100 mg/L) was allowed to proceed at 37°C for 10 min, and then the decrease in absorbance of the resulting solution was measured at 734 nm using a spectrophotometer (SPECTRONIC 200; Thermo Fisher Scientific Inc.). For the assay using DPPH radicals, the absorbance of DPPH radicals in 80% (*v*/*v*) aqueous methanol was set to 0.650 ± 0.020 at 517 nm using a spectrophotometer (SPECTRONIC 200; Thermo Fisher Scientific Inc.). DPPH radicals and PBE (100 mg/L) were reacted together at 23°C for 30 min. The absorbance of the resulting solution was monitored at 517 nm using a spectrophotometer (SPECTRONIC 200; Thermo Fisher Scientific Inc.).

2. Results and discussion

2.1. Total phenolic and flavonoid content

The total phenolic content of PBE was approximately 433.4 mg GAE/g (Table S1), which is similar to those of other bark extracts of *Pinus* species; 360.8 mg GAE/g for *P. pinaster* (maritime pine) [4], 550 mg GAE/g for *P. radiata* [5], and 299.3 mg GAE/g for *P. cembra* [6].

The total flavonoid content of PBE was approximately 299.4 mg CE/g (Table S1), which is similar to those of other bark extracts of the *Pinus* species; 379 mg CE/g for *P. durangensis* [7], 403 mg CE/g for *P. pinaster* [8], and 125.3 mg CE/g for *P. cembra* [6].

2.2. Antioxidant capacity

The antioxidant activities of PBE were approximately 540.5 and 402.6 mg VCE/g in the ABTS and DPPH assays, respectively (Table S1). A previous study showed that the bark of *P. densiflora* had a higher radical scavenging activity than the bark of *P. thunbergii* and *P. pinaster* (Pycnogenol®) in the ABTS assay [9]. Also, the results in this study were similar to those of another extract of *P. densiflora* bark extract (PineXol®) showing 697.3 mg VCE/g in the ABTS assay and 521.8 mg VCE/g in the DPPH assay [10]. The antioxidant capacity of PBE was due to phenolics in PBE such as procyanidins and their building blocks [(+)-catechin and (-)-epicatechin], and taxifolin and its derivatives, which were reported to have strong antioxidant capacities [11-14].

Table S1

Total phenolic and flavonoid content, and antioxidant capacity of *Pinus densiflora* Sieb. et Zucc. bark extract (PBE)

	**Total phenolic content (mg gallic acid equiv./g)**	**Total flavonoid content (mg catechin equiv./g)**	**Antioxidant capacity (mg vitamin C equiv./g)**	
			**ABTS** [a]	**DPPH** [b]
PBE	433.4 ± 9.0 [c]	299.4 ± 11.3	540.5 ± 8.2	402.6 ± 22.9

[a] 2,2-Azino-bis(3-ethylbenzothiazoline-6-sulfonic acid) radical scavenging assay

[b] 2,2-Diphenyl-1-picrylhydrazyl radical scavenging assay

[c] Data are experssed as means ± standard deviations (n = 3)

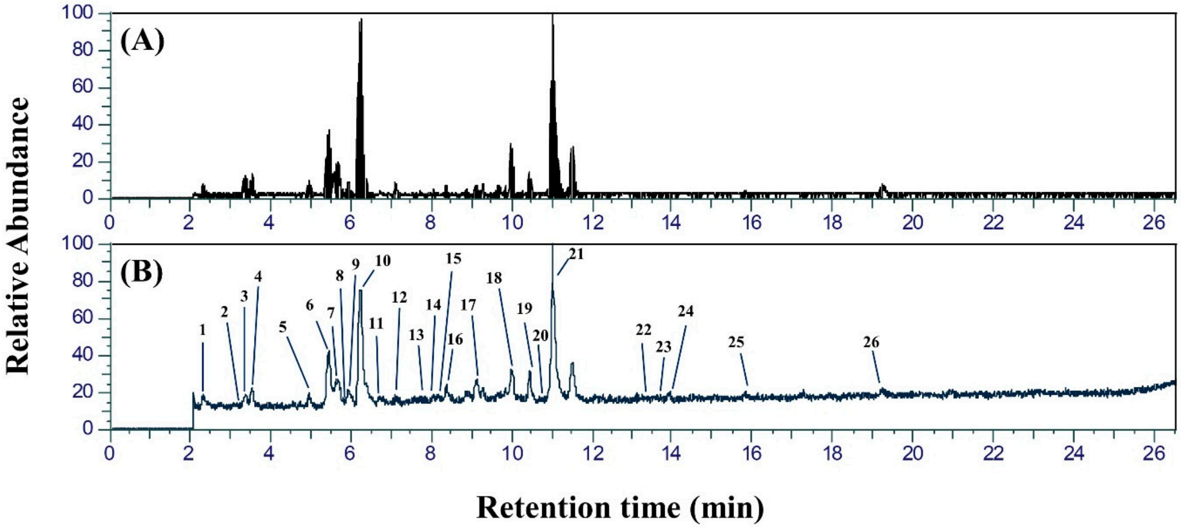

Figure S1. (A) Total ion chromatogram and (B) base peak chromatogram (filtered with MS[1]) of *Pinus densiflora* Sieb. et Zucc. bark extract (PBE). Peaks: 1, procyanidin trimer; 2, hydroxymandelic acid; 3, syringaldehyde; 4, protocatechuic acid; 5, unknown; 6, 3-*p*-coumaroylquinic acid; 7, procyanidin B1; 8, procyanidin B3; 9, procyanidin trimer; 10, (+)-catechin; 11, procyanidin trimer; 12, caffeic acid; 13, 5-*p*-coumaroylquinic acid; 14, (−)-epicatechin; 15, procyanidin trimer; 16, vanillyl ethyl ester hexoside; 17, procyanidin B2; 18, taxifolin 3-*O*-glucoside; 19, unknown; 20, luteolin; 21, taxifolin; 22, taxifolin isomer; 23, quercetin 3-*O*-rhamnoside; 24, dehydroxyltaxifolin; 25, quercetin 7-*O*-glucoside; 26, quercetin.

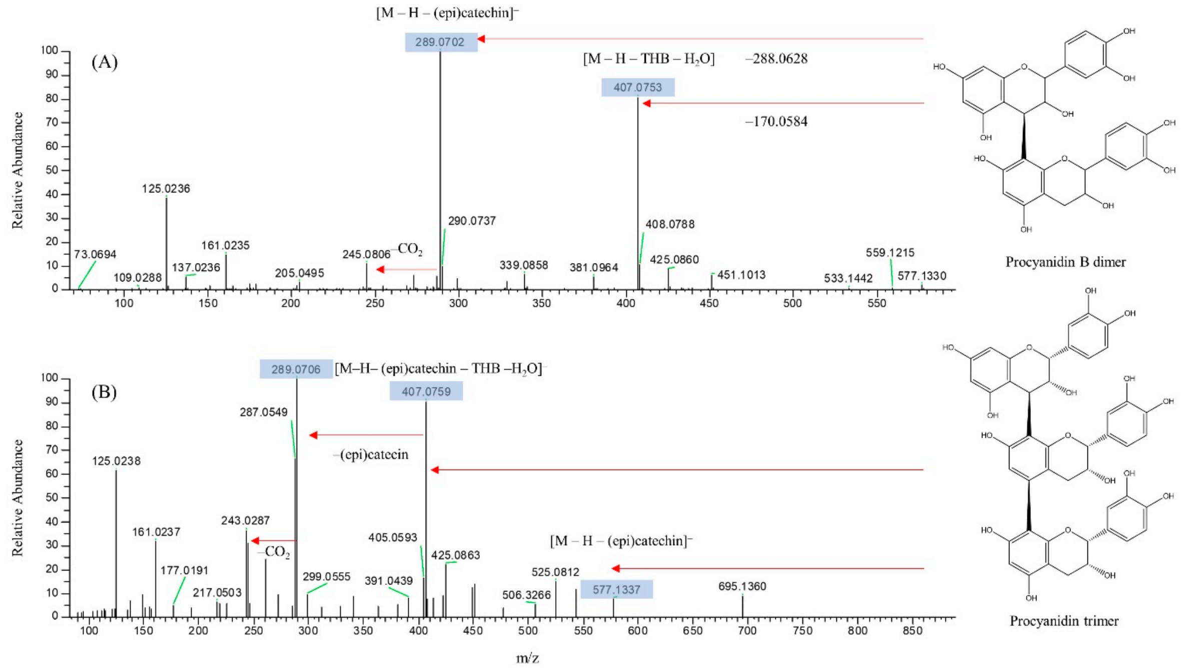

Figure S2. Typical fragmentation patterns for procyanidins (A) dimer and (B) trimer. The highlight *m*/*z* is characteristic MS[2] fragment ions.

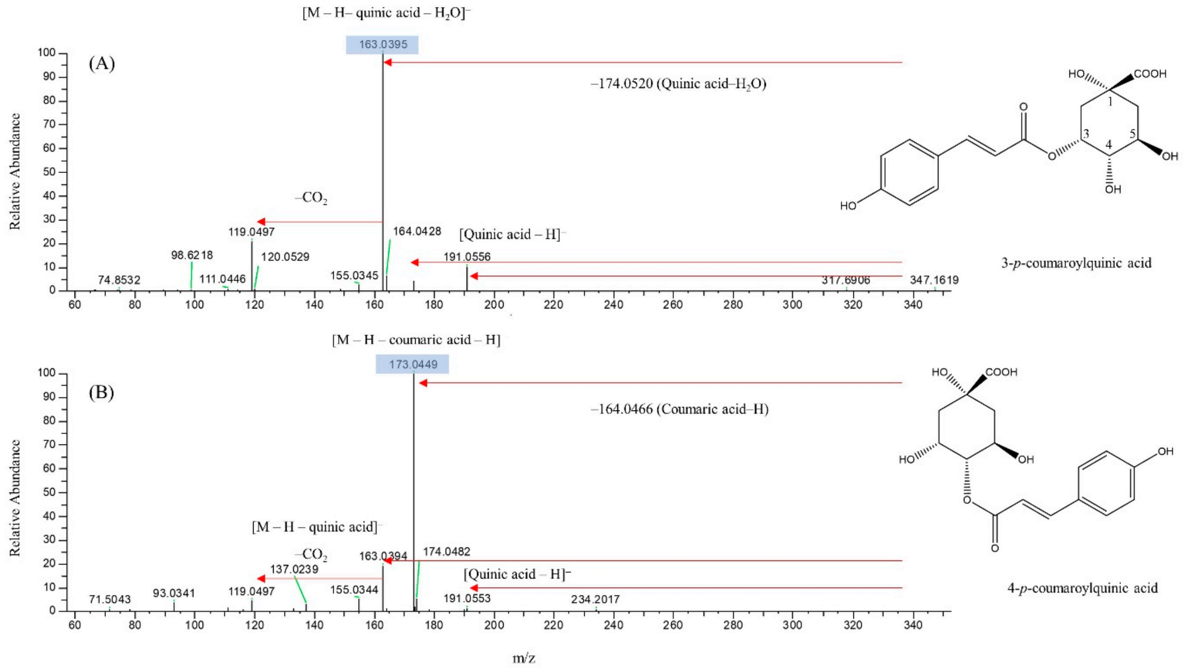

Figure S3. Typical fragmentation patterns for (A) 3-*p*-coumaroylquinic acid and (B) 5-*p*-coumaroylquinic acid. The highlight *m*/*z* is characteristic MS[2] fragment ions.

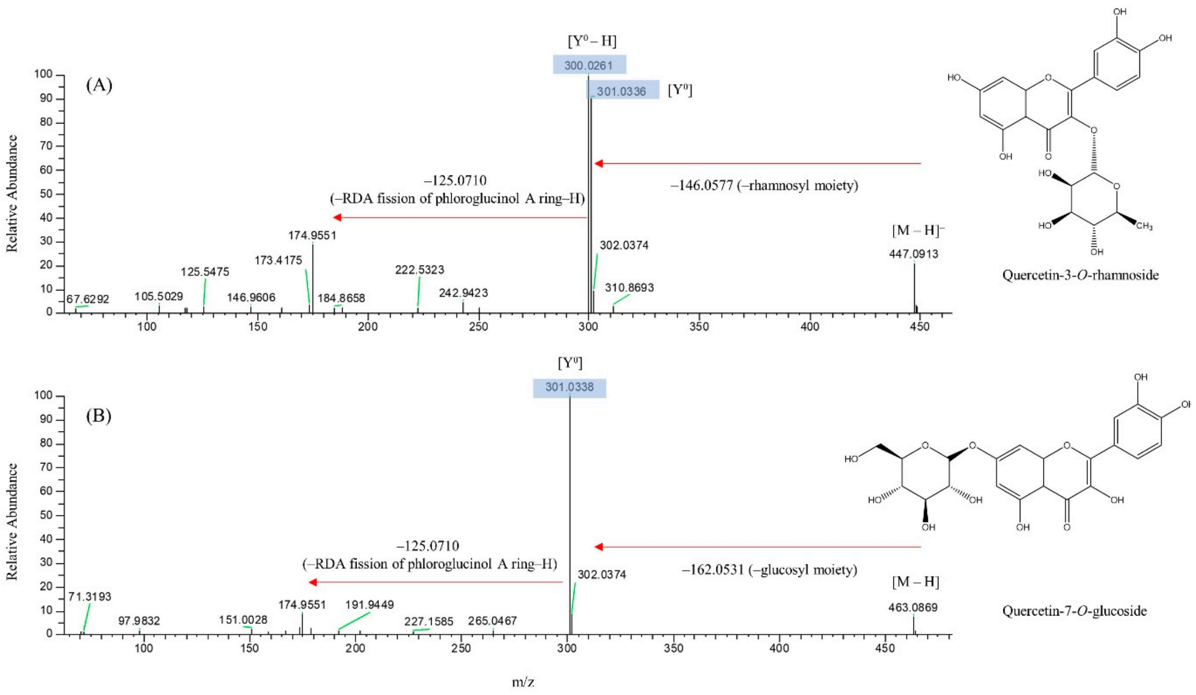

Figure S4. Typical fragmentation patterns for (A) quercetin 3-*O*-rhamnoside and (B) quercetin 7-*O*-glucoside. The highlight *m*/*z* is characteristic MS^2 fragment ions.

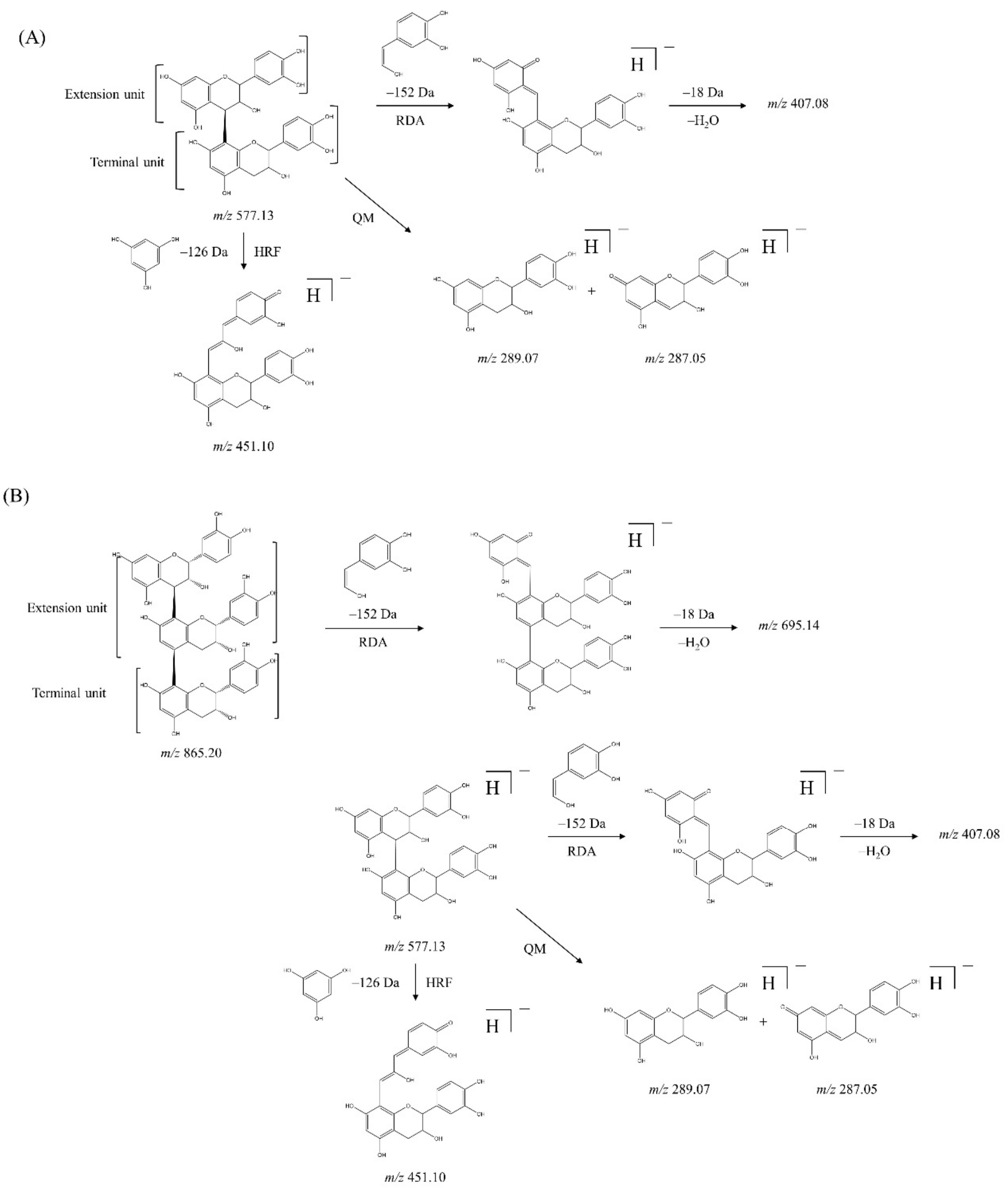

Figure S5. Typical fragmentation patterns for (A) procyanidin B type dimer and (B) procyanidin trimer via retro-Diels-Alder (RDA), heterocyclic ring fission (HRF), and quinone methide (QM) reactions.

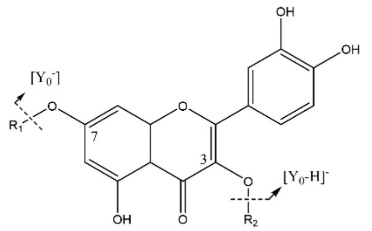

Figure S6. MS^2 characteristics of O-glycosylated hydroxyflavonol.

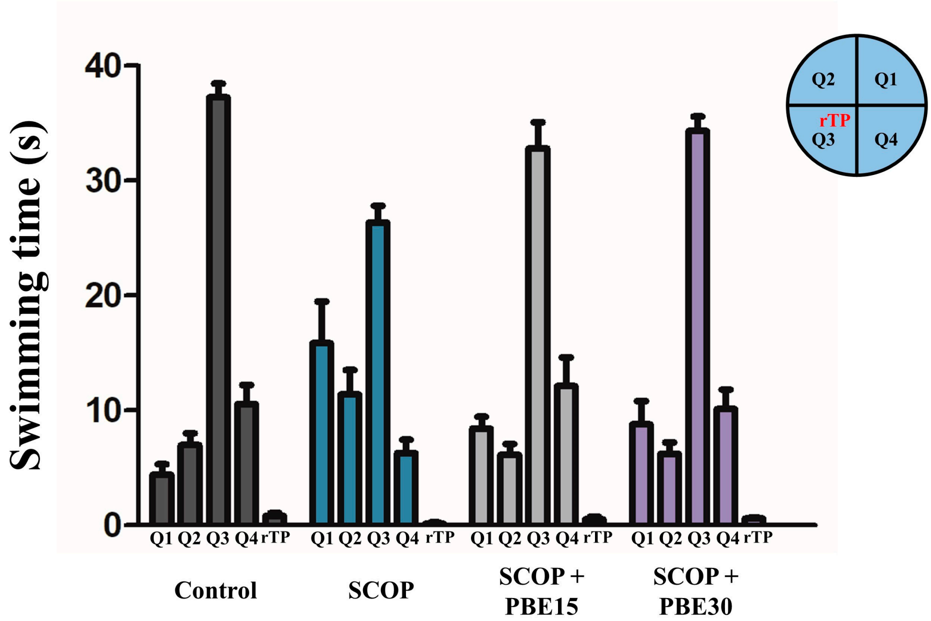

Figure S7. Swimming time in each quadrant of trial acquisition in the Morris water maze test. The removed target platform (rTP) is located in target quadrant 3. Quadrants: Q1, quadrant 1; Q2, quadrant 2; Q3, quadrant; Q4 quadrant 4.
